# Supplementary material for: Unified system describing factors related to the eradication of an alien plant species
Source: PeerJ. 2022 May 2;10:e13027. doi: 10.7717/peerj.13027 (PMC9070320; doi:10.7717/peerj.13027)
Supplement: Supplemental Information 1 — Evaluation of the importance of factors affecting the probability of eradication of: Poa annua L. from Antarctic King George Island, P. annua from Subantarctic Macquarie Island and Stellaria media (L.) Vill. from Macquarie Island. Factor’s impact on eradication success is scored using categories described in Table 2. [file peerj-10-13027-s001.pdf]

article title - **Unified system describing factors related to the eradication of an alien plant species**
journal name - **PeerJ**
authors - **Halina Galera, Agnieszka Rudak, Maciej Wódkiewicz**
corresponding author - **Agnieszka Rudak, a.rudak@biol.uw.edu.pl**

Supplemental Data S1

An exemplary application of the Unified System for assessing Eradication Feasibility (USEF): a comparison of eradication actions according to "one species / different regions" and "one region / different species" schemes.

Table S1:
Evaluation of eradication feasibility of: *Poa annua* L. from Antarctic King George Island (unless otherwise stated, data comes from *Galera et al. 2021*), *P. annua* from Subantarctic Macquarie Island and *Stellaria media* (L.) Vill. from Macquarie Island. For scoring and definitions of categories for scoring see Table 2, factor's input is calculated by dividing factor score by the factor's score maximum value (see text), **?** - information not available.

**Factor**	**Factor's input and rationale behind its evaluation for:**		
	***Poa annua* on King George Is.**	***Poa annua* on Macquarie Is.**	***Stellaria media* on Macquarie Is.**
1. Infestation size	**0.20**: minimum ≤ 5 m^2, maximum 0.76 ha (status for the 2017/2018 season)	**?**: information on the species distribution within the Island has not been published. However at present *P. annua* is described as "the most abundant non-native species" (*March-Salas & Pertierra, 2020*; see also *Williams et al., 2018, 2019a*)	**0.20**: 0.3 ha (*Williams et al., 2019b*)
2. Number of separate infestation sites	**0.40**: two infestation sites (status for the 2017/2018 season)	**?**: information on the species distribution within the Island has not been published. It is expected that the species displayed a high population dynamics due to the decreasing rabbit population (*Whinam et al., 2014*)	**0.80**: eight loosely defined subpopulations and six isolated individuals outside these subpopulations (*Williams et al., 2019b*)

**Factor**	***Poa annua* on King George Is.**	***Poa annua* on Macquarie Is.**	***Stellaria media* on Macquarie Is.**
3. Isolation of infestation	**0.20**: Island area – approximately 1300 km^2, the distance of the Island from South America – approximately 1000 km (*Korczak-Abshire, Angiel & Wierzbicki, 2011*); strong ecological isolation of the infestation within the Island (infestation located on the seasonally ice-free "environmental island" – Point Thomas Oasis with an area of 5.24 km^2)	**0.20**: Island area – 127.8 km^2 (*Macquarie Island Nature Reserve and World Heritage Area, 2006; Pertierra et al., 2016*), the distance of the Island from Australia – approximately 2000 km (*Distances [2020]*)	**0.20**: Island area – 127.8 km^2 (*Macquarie Island Nature Reserve and World Heritage Area, 2006; Pertierra et al. 2016*), the distance of the Island from Australia – approximately 2000 km (*Distances [2020]*)
4. Monitoring area size	**0.40**: monitoring area at initial stage of eradication 4.8 ha	**?**: information on the species distribution within the Island has not been published	**1.00**: current monitoring area – 1008 ha (*Williams et al., 2019b*)
5. Monitoring rate	**0.80**: number of required visits during the year – maximum 7<br>a) short annual monitoring period – growing season lasts maximum 3 months<br>b) recommended high monitoring frequency – 2 weeks	**?**: information on monitoring intensity has not been published	**?**: information on monitoring intensity has not been published; if pre-productive period of the species is the same as in other conditions, the area of infestation "will need to be monitored within a 5 week period" (*Williams et al*., 2019b)
6. Land use and ownership of infested area	**0.33**: no difficulties in accessing the target area - no ownership conflicts, legal regulations promote eradication<br>a) type of land management practices – extremely low intensity of land use<br>b) high accessibility resulting from land ownership relations – the whole maximum monitoring area managed by one Station operator<br>c) extremely low complexity of management mosaic	**0.33**: no difficulties in accessing the target area - no ownership conflicts, legal regulations promote eradication (*Macquarie Island Nature Reserve and World Heritage Area, 2006*)<br>a) type of land management practices – extremely low intensity of land use<br>b) high accessibility resulting from land ownership relations – the whole Island managed by one Station operator<br>c) extremely low complexity of management mosaic	**0.33**: no difficulties in accessing the target area - no ownership conflicts, legal regulations promote eradication (*Macquarie Island Nature Reserve and World Heritage Area, 2006*)<br>a) type of land management practices – extremely low intensity of land use<br>b) high accessibility resulting from land ownership relations – the whole Island managed by one Station operator<br>c) extremely low complexity of management mosaic

**Factor**	***Poa annua* on King George Is.**	***Poa annua* on Macquarie Is.**	***Stellaria media* on Macquarie Is.**
7. Accessibility	**0.20**:<br>a) very short distance to be traveled by personnel performing eradication: mean distance from the main Station building – 0.2 km (Station subpopulation) and 1.5 km (moraine subpopulation)<br>b) no difficulties in gaining access to infested area due to landform	**?**: information on the species distribution within the Island has not been published<br>a) the maximum possible distance to be traveled by personnel performing eradication is about 33 km (distance from the Station building to the farthest southern part of the Island, see Map 6 in *Macquarie Island Nature Reserve and World Heritage Area, 2006*), if the species occurs in the south of the island, then the southernmost sites will be difficult to access (no access by vehicle, few landing beaches, see Maps 4, 13 and 14 in *Macquarie Island Nature Reserve and World Heritage Area, 2006*)<br>b) topography of the island unfavorable for its exploration (Map 6 in *Macquarie Island Nature Reserve and World Heritage Area, 2006; Williams et al. 2018*), access may be difficult due to dense concentrations of seals and penguins (see Maps 9 and 11 in *Macquarie Island Nature Reserve and World Heritage Area, 2006*)	**0.80**:<br>a) distance to be traveled by personnel performing eradication moderate: max distance from the Station building to the farthest positions – about 8 km (see Fig. 1A by *Williams et al., 2019b*)<br>b) most sites located off established tracks, in steep terrain and creeks, access to some subpopulations is difficult due to dense concentrations of seals and penguins (*Williams et al., 2019b*)
8. Adaptation to new climatic conditions	**1.00**: climate adaptability high – an almost cosmopolitan species present in temperate, cold and polar zones (*Heide, 2001; Galera, Chwedorzewska & Wódkiewicz, 2015*); climate distance short - species present in the same polar climatic zone	**1.00**: climate adaptability high –an almost cosmopolitan species present in temperate, cold and polar zones (*Heide, 2001; Galera Chwedorzewska & Wódkiewicz, 2015*); climate distance short - species present in the same polar climatic zone	**1.00**: climate adaptability high – an almost cosmopolitan species present in temperate, cold and polar zones (*Sobey, 1981*); climate distance short - species present in the same polar climatic zone

**Factor**	***Poa annua* on King George Is.**	***Poa annua* on Macquarie Is.**	***Stellaria media* on Macquarie Is.**
9. Number and distribution of propagules	**0.40**: soil seed bank highly concentrated: around 6000 seeds m^{-2} under tussocks, 200-400 seeds m^{-2} in random places away from the tussocks	**1.00**: soil seed bank density spatially variable: 132,000 seeds m^{-2} at low altitude coastal sites, declined with increasing altitude to <2600 seeds m^{-2} (*Williams et al., 2016a*)	**1.00**: soil seed bank highly concentrated: mean density 3417 seeds m^{-2}, maximum 38,760 (or 37,740) seeds m^{-2} under canopy of *S. media*, and almost 0 just 60 cm away from the plants canopy (*Williams et al., 2019b*)
10. Vegetative propagation	**0.50**: very small ability of the plant to produce vegetative propagules (*Bond et al., 2007*)	**0.50**: very small ability of the plant to produce vegetative propagules (*Bond et al., 2007*)	**1.00**: vegetative propagation by fragmentation common – adventitious roots at nodes of prostrate stems, and on shoots severed from the parent plant (*Sobey, 1981*)
11. Propagule longevity	**1.00**: maximum longevity of seeds estimated at 16 years	**0.33**: “Seed viability declined over time, from an initial viability of 81 to <3% after 2 years in the soil” (*Williams et al., 2016a*)	**?**: “Mean viability declined from 92% to 79% after 12 months burial” (*Williams et al., 2019b*)
12. Pre-reproductive period	**1.00**: minimum length of the pre-reproductive period – few weeks (probably less than 1 month). Plants were also observed to start flowering under snow	**?**: not assessed on the Island	**0.80**: minimum length of the pre-reproductive period – probably 5 weeks (*Williams et al., 2019b*)
13. Detection possibility	**0.60**: species identifiable from a distance <2 m<br>a) detection distance 1-3 m (plants small, not uniquely coloured)<br>b) no specific smell, remote sensing not applicable (small plant)<br>c) target species similar to the native grass species	**0.60**: species identifiable from a distance <2 m<br>a) detection distance 1-3 m (plants small, not uniquely coloured)<br>b) no specific smell, remote sensing not applicable (small plant)<br>c) target species similar to the native grasses, including 3 *Poa* species (*Springer, 2012; Salas & Baker, 2019; March-Salas & Pertierra, 2020*) - this applies especially to juvenile plants	**0.60**: species identifiable from a distance <2 m (*Williams et al., 2019b*)<br>a) detection distance 1-3 m (plants small, but uniquely coloured)<br>b) the applicability of detector dogs is limited because of presence of native *Stellaria* species; remote sensing not applicable (small plant)<br>c) high possibility to distinguish target species from native plants visually

**Factor**	***Poa annua* on King George Is.**	***Poa annua* on Macquarie Is.**	***Stellaria media* on Macquarie Is.**
14. Annual period of detectability prior to seed set	**1.00**: probably 1-3 weeks (especially at the beginning of the growing season)	**?**: not assessed on the Island	**0.80**: probably longer than 5 weeks ("the gross area of infestation will need to be monitored within a 5 week period", *Williams et al., 2019b*)
15. Knowledge of current location of infestation sites	**0.33**: available information about current location of infected sites (distribution map and GPS coordinates published in a scientific journal, see *Galera et al., 2017*)	**1.00**: information on the species distribution within the Island has not been published	**0.33**: available information about current location of infected sites (distribution map published in a scientific journal, see *Williams et al., 2019b*)
16. Understanding of species biology	**0.33**: sufficient knowledge of target population biology to plan and execute eradication actions	**0.33**: sufficient knowledge of target population biology to plan and execute eradication actions "Aspects of the ecology of the species on the SOI [Southern Ocean Islands] are relatively well known" (*Williams et al., 2019a*)	**0.33**: sufficient knowledge of target population biology to plan and execute eradication actions (*Williams et al., 2019b*)
17. Eradication achieved elsewhere	**0.50**: successful eradication of several very small populations of the species in Maritime Antarctic has been reported	**0.75**: successful eradication of several very small populations of the species in Maritime Antarctic, but eradication attempt in similar conditions on South Ocean Snares Island failed (*Shaw, 2013*)	**1.00**: failed eradication attempt on South Ocean Snares Island (*Shaw, 2013*)
18. Reaction time	**1.00**: reaction time around 30 years	**1.00**: species first recorded in 1883 (*Pertierra et al., 2016*) or 1894 (*Copson & Whinam, 2001*), but the eradication has not yet started	**1.00**: species first recorded 1894 (*Copson & Whinam, 2001*), but the eradication has not yet started
19. Applicable control methods	**0.66**: available control measures:<br>a) only laborious and time consuming physical methods applicable and effective: hand weeding and hand removal of seed-infested soil<br>b) cultural control – N/A (no cultivations in Thomas Point Oasis)	**0.66**: available several control measures:<br>a) physical control was considered rather insufficient (*Williams et al., 2016b*)<br>b) cultural control – N/A (no cultivations on the Island, except for	**1.00**: effectiveness of control methods in the Subantarctic conditions requires research<br>a) difficult physical control due to soil seed bank and vegetative reproduction by fragmentation (*Williams et al., 2019b*)

Factor	*Poa annua* on King George Is.	*Poa annua* on Macquarie Is.	*Stellaria media* on Macquarie Is.
	c) possibility of using chemical methods limited (unknown impact on native species)<br>d) biological control methods cannot be used due to prohibited introduction of alien organisms	hydroponics; *Copson* & *Whinam, 2001; Macquarie Island Nature Reserve and World Heritage Area*, *2006*)<br>c) selective impact of glyphosate, rimsulfuron and trifloxysulfuron has been found under simulated Subantarctic temperatures (*Williams et al., 2016b, 2019a*)<br>d) biological control methods cannot be used due to prohibited introduction of alien organisms and the presence of a native three *Poa* species (*Macquarie Island Nature Reserve and World Heritage Area, 2006; Salas & Baker, 2019*)	b) cultural control – N/A (no cultivations on the Island, except for hydroponics, *Copson & Whinam, 2001; Macquarie Island Nature Reserve and World Heritage Area, 2006*)<br>c) the effectiveness and selectivity of chemical control methods in the Subantarctic conditions needs to be checked<br>d) biological control methods cannot be used due to prohibited introduction of alien organisms and the presence of a native *S. parviflora* (*Macquarie Island Nature Reserve and World Heritage Area, 2006; Salas & Baker, 2019*)
20. Personnel awareness	**0.66**: variable sense of responsibility of people involved in the campaign	**?**: no report on the start of eradication action available	**?**: no report on the start of eradication action available
21. Coordination between monitoring agencies	**0.75**: during the eradication action the set of institutions coordinating the project and personal composition of eradication team changed significantly over time with varying support of qualified personnel	**?**: no report on the start of eradication action available	**?**: no report on the start of eradication action available.
22. Sufficient allocation of resources	**0.50**: financing less than sufficient, but stable; little additional resources needed for the campaign apart from Polish Antarctic Station maintenance cost	**?**: the eradication action has not started	**?**: the eradication action has not started

**Factor**	***Poa annua* on King George Is.**	***Poa annua* on Macquarie Is.**	***Stellaria media* on Macquarie Is.**
23. Economic and social relevance of target species	**0.33**: no conflicts with the public:<br>a) no cultivation value<br>b) pressure on eradication due to legal conditions	**0.33**: no conflicts with the public:<br>a) no cultivation value<br>b) pressure on eradication due to legal conditions (*Macquarie Island Nature Reserve and World Heritage Area, 2006*)	**0.33**: no conflicts with the public:<br>a) no cultivation value<br>b) pressure on eradication due to legal conditions (*Macquarie Island Nature Reserve and World Heritage Area, 2006*)
24. Invasion pathways	**0.33**: potential pathways of invasion limited and at least partially blocked by phytosanitary regulations:<br>a) proven propagule pressure of the species on the Island (*Lityńska-Zając et al., 2012*). Site relevant drivers known to increase invasion risk in the broader Antarctic described by *McGeoch et al.* (*2015*) such as: scientific activity, tourism, year-long residents, importation of fresh produce, airfield (helipad). There is no agriculture on the Island.<br>b) methods of blocking pathways of invasion available (*Non-Native Species Manual, 2019*)	**0.33**: potential pathways of invasion limited and at least partially blocked by phytosanitary regulations:<br>a) proven propagule pressure of the species on the Island (*Whinam, Chilcott & Bergstrom, 2005*). Site relevant drivers known to increase invasion risk in the broader Antarctic described by *McGeoch et al.* (*2015*) such as: scientific activity, tourism, year-long residents, importation of fresh produce, airfield (helipad), agriculture (only hydroponics, set of plants that are allowed for cultivation) (*Pertierra et al., 2016; Macquarie Island Nature Reserve and World Heritage Area, 2006*)<br>b) methods of blocking pathways of invasion available (*Whinam, Chilcott & Bergstrom, 2005; Pertierra et al., 2016*), effective and thoroughly applied strict quarantine and environmental protection measures (*Macquarie Island Nature Reserve and World Heritage Area, 2006*)	**0.33**: potential pathways of invasion limited and at least partially blocked by phytosanitary regulations:<br>a) no proven propagule pressure of the species (*Whinam, Chilcott & Bergstrom, 2005*), however *S. media* is one of the most common invasive species in the Subantarctic (*Williams et al., 2019b*). Site relevant drivers known to increase invasion risk in the broader Antarctic described by *McGeoch et al.* (*2015*) such as: scientific activity, tourism, year-long residents, importation of fresh produce, airfield (helipad), agriculture (only hydroponics, set of plants that are allowed for cultivation) (*Macquarie Island Nature Reserve and World Heritage Area, 2006; Pertierra et al., 2016*)<br>b) methods of blocking pathways of invasion available (*Whinam, Chilcott & Bergstrom, 2005; Pertierra et al., 2016*), effective and thoroughly applied strict quarantine and environmental protection measures (*Macquarie Island Nature Reserve and World Heritage Area, 2006*)

REFERENCES

Bond W, Davies G, Turner R. 2007. The Biology and Non-chemical Control of Annual Meadow-Grass *(Poa annua* L.). *Available at http://www.gardenorganic.org.uk/weeds/annual-meadow-grass* (accessed on 24 May 2017).

Copson G, Whinam J. 2001. Review of ecological restoration programme on subantarctic Macquarie Island: pest management progress and future directions. *Ecol Manag Restor* **2:**129–138 DOI: 10.1046/j.1442-8903.2001.00076.x.

Distances [2020]. The distance in kilometres between various cities and Australia's Antarctic stations and subantarctic islands. In: Australian Antarctic Programm. Kingston: Department of Agriculture, Water and the Environment of Australian Government. *Available at https://www.antarctica.gov.au/about-antarctica/geography-and-geology/geography/distances/* (accessed on 8 January 2022).

Galera H, Chwedorzewska KJ, Wódkiewicz M. 2015. Response of *Poa annua* to extreme conditions: comparison of morphological traits between populations from cold and temperate climate conditions. *Polar Biol* **38**:1657–1666 DOI: 10.1007/s00300-015-1731-y.

Galera H, Wódkiewicz M, Czyż E, Łapiński S, Kowalska ME, Pasik M, Rajner M, Bylina P, Chwedorzewska KJ. 2017. First step to eradication of *Poa annua* L. from Point Thomas Oasis (King George Island, South Shetlands, Antarctica). *Polar Biol* **40**:939–945 DOI: 10.1007/s00300-016-2006-y.

Galera H, Znój A, Chwedorzewska KJ, Wódkiewicz M. 2021. Evaluation of factors influencing the eradication of annual bluegrass (*Poa annua* L.) from Point Thomas Oasis, King George Island, Maritime Antarctica. *Polar Biol* **44**:2255–2268 DOI: 10.1007/s00300-021-02941-1.

Heide OM. 2001. Flowering responses of contrasting ecotypes of *Poa annua* and their putative ancestors *Poa infirma* and *Poa supina*. *Ann Bot* **87**:795–804.

Korczak-Abshire M, Angiel PJ, Wierzbicki G. 2011. Records of white-rumped sandpiper (*Calidris fuscicollis*) on the South Shetland Islands. *Polar Rec* **47**:262–267 DOI: 10.1017/S0032247410000665.

Lityńska-Zając M, Chwedorzewska KJ, Olech M, Korczak-Abshire M, Augustyniuk-Kram A. 2012. Diaspores and phyto-remains accidentally transported to the Antarctic Station during three expeditions. *Biodivers Conserv* **21**:3411–3421 DOI: 10.1007/s10531-012-0371-6.

Macquarie Island Nature Reserve and World Heritage Area. 2006. Management Plan 2006. Hobart: Parks and Wildlife Service, Department of Tourism, Arts and the Environment. *Available at https://parks.tas.gov.au/Documents/Macquarie%20Island%20Nature%20Reserve%20and%20World%20Heritage%20Area%20Management%20Plan%202006.pdf* (accessed on 14 April 2021).

March-Salas M, Pertierra LR. 2020. Warmer and less variable temperatures favour an accelerated plant phenology of two invasive weeds across sub-Antarctic Macquarie Island. *Austral Ecol* **45**:572–585 DOI: 10.1111/aec.12872.

McGeoch MA, Shaw JD, Terauds A, Lee JE, Chown SL. 2015. Monitoring biological invasion across the broader Antarctic: A baseline and indicator framework. *Glob Environ Chang* **32**:108–125 DOI: 10.1016/j.gloenvcha.2014.12.012.

Non-Native Species Manual. 2019. Revision 2019. Secretariat of the Antarctic Treaty, Buenos Aires. *Available at https://documents.ats.aq/ATCM42/WW/ATCM42_WW008_e.pdf* (accessed on 22 April 2021).

Pertierra LR, Baker M., Howard C, Vega GC, Olalla-Tarraga MA, Scott J. 2016. Assessing the invasive risk of two non-native *Agrostis* species on sub-Antarctic Macquarie Island. *Polar Biol* **39**:2361–2371 DOI: 10.1007/s00300-016-1912-3.

de Salas MF, Baker ML. 2019. A Census of the Vascular Plants of Tasmania, including Macquarie Island. Tasmanian Herbarium, Tasmanian Museum and Art Gallery, Hobart. *Available at https://www.tmag.tas.gov.au/__data/assets/pdf_file/0005/195800/2019_Census_of_Tasmanian_Vascular_Plants_-_PDF.PDF* (accessed on 14 April 2021).

Shaw JD. 2013 Southern Ocean Islands Invaded: Conserving Biodiversity in the World's Last Wilderness. In: Foxcroft LC, Pyšek P, Richardson DM, Genovesi P, eds. *Plant Invasions in Protected Areas: Patterns, Problems and Challenges, Invading Nature*. Springer Series in Invasion Ecology 7, Dordrecht: Springer, 449–470 DOI: 10.1007/978-94-007-7750-7_20.

Sobey D. 1981. *Stellaria media* (L.) Vill. Biological Flora of the British Isles 150. *J Ecol* **69**:311–335.

Springer K. 2012. Ecological restoration of sub-Antarctic Macquarie Island. In: Timm RM, ed. *Proceedings of the 25th Vertebrate Pest Conference.* Davis: University of California, 34–37 DOI: 10.5070/V425110513.

Whinam J, Chilcott N, Bergstrom DM. 2005. Subantarctic hitchhikers: expeditioners as vectors for the introduction of alien organisms. *Biol Conserv* **121**:207–219 DOI: 10.1016/j.biocon.2004.04.020.

Whinam J, Fitzgerald N, Visoiu M, Copson G. 2014. Thirty years of vegetation dynamics in response to a fluctuating rabbit population on sub-Antarctic Macquarie Island. *Ecol Manag Restor* **15**:41–51 DOI: 10.1111/emr.12076.

Williams L, Kristiansen P, Sindel BM, Wilson S, Shaw J. 2016a. Quantifying the seed bank of an invasive grass in the sub-Antarctic: seed density, depth, persistence and viability. *Biol Invasions* **18**:2093–2106 DOI 10.1007/s10530-016-1154-x.

Williams LK, Kristiansen P, Sindel BM, Shaw JD, Wilson1 SC. 2016b. Management of invasive *Poa annua* in the sub-Antarctic wilderness of Macquarie Island. Science, Community and Food Security: the Weed Challenge. In: *19th Australasian Weeds Conference, Hobart, 1-4 September 2014*. Palmerston North: Council of Australasian Weed Societies, 324–327.

Williams LK, Shaw JD, Sindel BM, Wilson SC, Kristiansen P. 2018. Longevity, growth and community ecology of invasive *Poa annua* across environmental gradients in the subantarctic. *Basic Appl Ecol* **29**: 20–31 DOI: 10.1016/j.baae.2018.02.003.

Williams LK, Sindel BM, Kristiansen P, Wilson SC, Shaw JD. 2019a. Assessing the efficacy and impact of management of an invasive species in a protected area: *Poa annua* on sub-Antarctic Macquarie Island. *Weed Res* **59**:180–190 DOI: 10.1111/wre.12355.

Williams LA, Fergus AJ, Shaw JD, Terauds A, Kristiansen P, Wilson SC, Gosden JL, Ziegler K, Sindel BM. 2019b. Quantifying site and species factors to inform the feasibility of eradication of alien plants from Southern Ocean Islands: *Stellaria media* on Macquarie Island. *Biol Invasions* **21**:993–1005 DOI:10.1007/s10530-018-1880-3.
